# Preconception allergen sensitization can induce B10 cells in offspring: a potential main role for maternal IgG

**DOI:** 10.1186/s13223-017-0195-8

**Published:** 2017-04-17

**Authors:** Marília Garcia de Oliveira, Luana de Mendonça Oliveira, Aline Aparecida de Lima Lira, Fábio da Ressureição Sgnotto, Alberto José da Silva Duarte, Maria Notomi Sato, Jefferson Russo Victor

**Affiliations:** 1 Laboratory of Medical Investigation LIM-56, Division of Clinical Dermatology, Medical School, University of Sao Paulo, Av. Dr. Enéas de Carvalho Aguiar, 500, 3rd Floor, 05403-000 Sao Paulo, Brazil; 2 Division of Pathology, Medical School, University of Sao Paulo, Sao Paulo, Brazil

**Keywords:** Allergy, Allergen-specific IgG, B10 cells, IL-10, Maternal immunization

## Abstract

**Background:**

The mechanisms through which allergies can be inhibited after preconception immunization with allergens are not fully understood. We aimed to evaluate whether maternal immunization can induce a regulatory B (B10) cell population in offspring in concert with allergy inhibition.

**Methods:**

C57BL/6 females were or were not immunized with OVA and were mated with normal WT males. Their offspring were evaluated at 3 days of age or 20 days after neonatal immunization. Human peripheral B cells from atopic and non-atopic individuals were also evaluated.

**Results:**

Preconception OVA immunization induced B10 cells in offspring, and IL-10 production appeared to be critical for FcγRIIB upregulation in offspring B cells. Murine and human IL-10-producing B cells responded in vitro to IgG according to the atopic repertoire of the cells.

**Conclusions:**

Our results reveal that maternal immunization induces allergen-specific B10 cells in offspring and a pivotal role for the IgG repertoire in IL-10 production by murine and human B cells.

**Electronic supplementary material:**

The online version of this article (doi:10.1186/s13223-017-0195-8) contains supplementary material, which is available to authorized users.

## Background

Our group has studied type I hypersensitivity inhibition in murine models for the last decade [1–7]. During this period, we proposed that maternal IgG can play a pivotal role in offspring immune modulation, but the mechanisms underlying this phenomenon were not fully elucidated. In the 90s, it was revealed that maternal antibodies are passively transferred to offspring during the gestational and weaning periods. These antibodies, particularly IgG, can interact directly with the offspring’s immune system, even in the absence of antigens [8], and might involve maternal antibody-allergen immune complexes that directly interact with the inhibitory receptor FcγRIIb (CD32b) expressed by offspring B cells [9]. These interactions can in turn regulate the production of IgE antibodies, thus inhibiting the development of allergies. The alterations in the offspring immune system that occur as a consequence of maternal IgG interactions are not fully understood. Because the main event of allergy inhibition is the control of IgE production by B cells, we believe that this population plays a pivotal role as the subject of the effects induced by maternal immunization.

Some populations of B cells can acquire regulatory properties (Breg), and among these cell populations, regulatory B (B10) cells, which have been identified in humans and mice, have high regulatory potential [10]. This population is characterized by a CD19^+^IL10^+^CD1d^high^ phenotype and high IL-10 secretion. The regulatory potential of B10 cells can inhibit OVA-induced allergic pulmonary inflammation [11]. The mechanism through which allergen-specific B10 cells can be induced and a possible role of IgG in this mechanism have not been described.

The aim of this study was to determine whether maternal immunization can induce regulatory B10 cells in offspring as a mechanism of allergy inhibition. Furthermore, we evaluated the importance of maternal IgG for the induction of B10 cells and whether evidence of similar mechanisms can be detected in humans.

## Methods

### Mice

C57BL/6 inbred wild-type (WT) or IL-10-genetically-deficient male and female mice were used at 8–10 weeks of age. The animals were purchased through the Central Animal Facility of the School of Medicine and Institute of Biomedical Sciences—USP. The offspring (F1) of both sexes were evaluated during the neonatal period, and samples from at least three independent experiments were studied.

### Patient samples

Peripheral blood mononuclear cells (PBMCs) and sera were collected from volunteers who were previously classified as atopic or non-atopic individuals according to their clinical status and who voluntarily submitted to a skin prick test (SPT) to confirm their atopic state. These individuals were classified into two groups: atopic individuals [clinically allergic and reactive to at least two allergens, n = 14, age (mean ± SE) = 31.1 ± 2.86] and non-atopic individuals [without any clinical allergy symptoms and unreactive to any tested allergen, n = 14, age (mean ± SE) = 28.2 ± 3.11].

Each PBMC sample was provided by a different donor and analyzed in three independent experiments.

### SPT and collection of blood samples

The subjects voluntarily submitted to a SPT according to European standards [12] with an adapted panel of allergens that included the Brazil pattern (*Blomia tropicalis, Canis familiaris, Periplaneta americana, Aspergillus fumigatus, Penicillium notatum, Alternaria alternata, Cladosporium herbarum, Dermatophagoides pteronyssinus, Dermatophagoides farinae,* and *Felis domesticus*).

In brief, a drop of each allergen extract, histamine (positive control) or allergen diluent (negative control—IPI-Asac, Brazil) was applied to the volar aspect of the forearm. A superficial skin puncture was made through each allergen or control drop with a hypodermic lancet (Alko, Brazil) without inducing bleeding. Fifteen minutes after puncture, the transverse diameter of each wheal reaction was measured. We considered a reaction to be positive when the wheal was 3 mm greater than the wheal diameter of the negative control. As exclusion criteria, we adopted the use of antihistamines, glucocorticosteroids and some other systemic drugs that can influence the results 15 days before the test. We also excluded volunteers with severe eczema or dermographism.

### Purification of mouse and human IgG

IgG antibodies were purified from the sera of mice that were immunized with OVA (40 days after immunization) or of non-immunized mice using a Melon Gel IgG Spin Purification kit, according to the manufacturer’s instructions (Thermo, USA). In brief, 500 μL of purification gel was placed in a mini column attached to a microtube, and the mixture was centrifuged for 1 min at 2000*g*. After the supernatant was discarded, 300 μL of washing buffer from the kit was added, and the resulting mixture was centrifuged again. The sample of pooled sera was added to the gel, homogenized for 5 min, and centrifuged, and the supernatant (purified IgG) was collected and stored at −70 °C for subsequent use in culture experiments.

To purify human IgG, two blood samples were obtained by venipuncture from each atopic or non-atopic individual in tubes without anticoagulants. After the blood samples were centrifuged at 1400*g* for 10 min, the serum was fractionated and pooled. Human IgG was purified using the Melon Gel IgG Spin Purification kit as described above, and the purified IgG was stored at −70 °C for subsequent use in culture experiments.

Both purified IgGs were sterilized using 0.20-micron filters (Corning, Germany), and their IgG concentrations were determined with the Coomassie Protein Assay Reagent (Pierce, USA) according to the manufacturer’s instructions.

### Murine immunization

Female WT mice were immunized subcutaneously with 6 mg of Alum (FURP, Sao Paulo) only or supplemented with 1500 μg of OVA (EndoFit™—endotoxin levels <1 EU/mg; InvivoGen, San Diego, CA, USA), 100 μg of myelin oligodendrocyte glycoprotein (MOG) peptide fragment 35–55 (Sigma, USA) or 100 μg of Dp (LoTox—Indoor Biotechnologies, USA). These animals where boosted intraperitoneally (i.p.) after 10 and 20 days with the same immunization antigen at the following doses: 1000 μg of OVA, 100 μg of MOG or 100 μg of Dp in saline. Thee females that were immunized with Alum only were boosted with saline alone. All females were mated 21 days post-immunization. The pups were maintained with their respective mothers during the breast-feeding period. Some groups of offspring from the immunized and non-immunized mothers were immunized with the same antigen used for maternal immunization. Specifically, 3-day-old offspring were treated i.p. with 100 μg of OVA or 10 μg of Dp in 0.6 mg of Alum and boosted after 10 days with the same antigen/dose in saline. Experimental analyses of the offspring were performed at 20 days of age. The OVA immunization protocols were also performed using IL-10^−/−^ or IL-10^−/+^ mice.

### Determination of the total IgE and anti-OVA IgG1/IgM antibody levels

The OVA-specific IgG1, IgM and total IgE antibodies were measured by ELISA as previously described [13]. To measure the total IgE level, a standard curve was used (Pharmingen, USA). The anti-OVA and anti-Dp Ab levels are expressed as optical densities. The anaphylactic anti-OVA IgE titer was measured through passive cutaneous anaphylaxis (PCA) as previously described [7].

### Murine lung inflammation

The offspring from either immunized or non-immunized mothers were immunized and nasally administered 100 μg of OVA (InvivoGen, San Diego, CA, USA) at 43, 50, 57, 58 and 59 days of age. The bronchoalveolar fluid (BAL) was analyzed at 60 days of age following exsanguination of the abdominal aorta. The BAL was obtained by washing the lungs three times with 1.5 mL of PBS using a tracheal tube followed by centrifugation at 2000*g* for 10 min. The cell pellet was diluted in 300 μL of PBS, and the total leukocyte counts were performed using a Neubauer chamber. Sections were cut at a thickness of 3 µm, mounted on slides and stained with HE for morphological analyses. The lungs were surgically collected and subjected to a tissue dissociation protocol.

### Spleen cell suspensions

The spleens were collected, and their cells were isolated for culture or flow cytometry analyses. Single-cell suspensions were prepared with cell strainers (BD Biosciences, MA, USA) and placed in Petri dishes containing RPMI 1640 culture medium (Sigma, USA). The cell suspension was treated with lysis buffer (Biosource—ACK Lysis Buffer, Rockville, MD, USA) for 2 min, and the cell suspension was washed twice with RPMI medium. The cells were subsequently resuspended in 1 mL of RPMI medium with 10% FBS (III HyClone, Logan, UT, USA), and the cellular viability was quantified with 0.5% Trypan blue in a Neubauer chamber.

### Murine flow cytometry

For surface staining, single-cell suspensions were prepared in flow cytometry buffer (PBS, 1% BSA). Anti-CD19 (1D3), anti-B220 (RA3-6B2), anti-CD1d (1B1), anti-IgM (R6-60.2) or anti-CD16/32 (2.4G2) antibodies directly conjugated to Cy-Chrome, PE, APC, Horizon V450, PE-Texas Red, PerCP, PerCP-Cy5 or FITC (BD Biosciences) were used at their optimal concentrations, as determined through titration experiments. Cell gating was based on the specific isotype control values as well as the fluorochrome minus 1 (FMO) setting when needed. The gating strategy for B cells that produce IL-10 and B10 cells is illustrated in Additional file 1: Figure S1. All analyses were performed using FlowJo software (Tree Star Inc.). For intracellular staining, the cells were first surface-stained, fixed (PBS, 1% formaldehyde—Sigma), permeabilized (PBS, 0.5% Saponin—Sigma), and subjected to intracellular staining with IL-10 (JES5-16E3) or a matching isotype. For the evaluation of intracellular cytokines, the cells were cultured for 24 h at 3 × 10^6^ cells/mL in RPMI 1640 (Gibco) supplemented with 10% heat-inactivated FCS (HyClone) without stimulus in the presence of 10 mg/mL brefeldin A during the last 12 h (Sigma-Aldrich). After the staining of surface markers, the cells were fixed with 4% formaldehyde in PBS (Merck) and then subjected to intracellular staining. Instrument compensation was performed using micro beads adsorbed with anti-rat/anti-hamster antibodies (CompBeads, BD Biosciences) and their conjugated antibodies. The acquisition of 50,000 events per sample was performed in the lymphocyte quadrant (as determined by the ratio of size to granularity) on an LSRFortessa cytometer (BD Biosciences, USA), and data analysis was performed using FlowJo software 7.6.5 (Tree Star, USA).

For the measurement of serum cytokines, the Th1/Th2/Th17A mouse CBA kit (Cytometric Bead Assay, BD Biosciences) was used according to the manufacturer’s instructions. In brief, microspheres of different fluorochrome intensities were sensitized with anti-IL-6 and anti-IL-10 and incubated either with the serum or supernatant for the generation of a standard curve or in the presence of capture antibodies for the same cytokine conjugated to PE for 2 h at room temperature. After washing with buffer supplied by the manufacturer, the microspheres were analyzed with an LSRFortessa cytometer (BD Biosciences, USA), and the levels were determined using CBA Analysis Software.

The BAL was prepared in PBS, and red blood cells were lysed using ammonium chloride lysis buffer. The BAL cells were stained as described by van Rijt et al. [14] with the optimal concentrations of the following monoclonal antibodies provided by BD Biosciences (San Diego, CA, USA): MHCII (2G9), CCR3 (101.111), CD11c (HL3), CD3 (145-2C11) and B220 (RA3-6B2). To prevent nonspecific binding to Fc receptors, a 2.4 G2-blocking reagent (6 µg/mL) was added to the monoclonal antibody mix. Lymphocytes were identified as FSClo/SSClo cells expressing CD3 or B220, and B cells were distinguished from T cells through MHCII expression in the (B220/CD3)^+^ gate. Granulocytes were recognized as non-autofluorescent highly granular (SSChi) cells. Within this gate, eosinophils were defined as cells that expressed the eotaxin receptor CCR3, intermediate levels of CD11c, and very low to undetectable levels of MHCII, B220 and CD3. Neutrophils presented a similar scatter profile to eosinophils but lacked CCR3 expression. Dendritic cells were identified as (CD3/B220)^+^ cells and expressed high levels of MHCII and CD11c. Alveolar macrophages were identified as large autofluorescent cells.

### Lung tissue dissociation

The lungs were collected through enzymatic dissociation and placed in 15-mL propylene conical centrifuge tubes. An enzymatic solution (2 mL) consisting of RPMI medium pre-warmed to 37 °C, 1 mg/mL collagenase D, 2 mg/mL DNase I (Roche Diagnostics, Mannheim, Germany), and 5% fetal bovine serum (FBS) was then added, and the sample was incubated for 15 min at 37 °C under continuous agitation. The digested tissue was homogenized gently and filtered through a plastic sieve with a 70-μM mesh screen (Cell Strainer, BD Falcon, CA, USA) to remove aggregates. The resultant cell suspensions were washed twice with 10 mL of RPMI medium (Gibco—Life Technologies, NY, USA) pre-warmed to 37 °C and then centrifuged at 500*g* for 5 min.

### Murine cell culture

To investigate the FcγRIIb expression kinetics in B cells, the phenotypes of 3 × 10^6^ cells/mL normal adult splenic B cells were evaluated by flow cytometry ex vivo or after culture for 24, 48, 72 or 96 h in RPMI 1640 (Gibco) supplemented with 10% heat-inactivated FCS (HyClone) in the presence of 50 μg/mL anti-IgM F(ab’)2 (Southern Biotech, USA).

To investigate the in vitro effect of purified maternal antibodies on offspring B cells, splenocytes from 3-day-old offspring of immunized or non-immunized mothers were cultured for 120 h at a density of 3 × 10^6^ cells/mL in RPMI 1640 (Gibco) supplemented with 10% heat-inactivated FCS (HyClone) in the presence of 20 μg/mL OVA (Grade V—Sigma, St. Louis, MO, USA) or 100 μg/mL purified IgG from immunized or non-immunized mothers. Some culture wells were previously blocked with 10 μg/mL purified anti-CD16/32 (2.4G2). For the evaluation of intracellular cytokine production, all of the culture wells were given 10 mg/mL brefeldin A (Sigma-Aldrich) 24 h before flow cytometric evaluation.

### Separation of PBMCs

To obtain PBMCs from atopic or non-atopic individuals, blood samples of approximately 20 mL were collected by venipuncture in sodium heparin-containing tubes. The blood was diluted in physiological saline at a 1:1 proportion of blood to saline. The blood was then placed in conical tubes containing Ficoll-Paque Plus (GE Healthcare, Sweden) at a proportion of 1:3. This material was centrifuged at 800*g* for 20 min, and the cells were subsequently washed twice in saline and resuspended in 1 mL of RPMI 1640 containing 10% fetal bovine serum (FBS). A sample of this suspension was diluted at a ratio of 1:2 in Trypan blue for assessments of cell viability and counts under an optical microscope using a Neubauer chamber.

### Human cell culture and flow cytometry

Thawed, freshly separated PBMCs were washed twice with 10 mL of RPMI medium at 37 °C, centrifuged at 800*g* for 10 min, and resuspended in 1 mL of RPMI 1640 medium containing 10% fetal bovine serum. A sample of this suspension was diluted 1:2 in Trypan blue (Sigma, USA) for evaluation of the cell viability and count under an optical microscope using a Neubauer chamber (Laboroptik, Germany). After dilution, 1 × 10^6^ viable PBMCs were placed in each well of a culture plate and cultured with 100 μg/mL IgG, which was purified from pools of atopic or non-atopic individuals in RPMI 1640 medium containing 10% fetal bovine serum in a total volume of 200 μL. The culture plate was incubated at 37 °C with 5% CO_2_ for 6 days, which was the period determined based on the kinetic assessment. After incubation, 1 mg/mL brefeldin A was added (Sigma, Israel) to each well of the culture plate, and flow cytometry labeling was performed after 24 h.

For extracellular staining, PBMCs at a concentration of 0.5 × 10^6^ were transferred to test tubes, and 1 μL of each antibody was added to the cells (except the unlabeled tubes) and incubated for 30 min at 4 °C while protected from light. After 500 μL of 1X PBS solution was added, the tubes were centrifuged at 800*g* for 5 min. The supernatant was discarded by inversion, and 300 μL of 1X PBS was added. For fixation, 200 μL of 1% formaldehyde was added for at least 10 min. The PBMCs were stained with mouse anti-human-CD4 conjugated to FITC or APC, anti-human-CD8 conjugated to PE and anti-human-CD19 PE-Cy7 antibodies (BD Pharmingen, NJ, USA) for the identification of B cells (CD4^−^CD8^−^CD19^+^).

For intracellular labeling, the tubes were centrifuged at 800*g* for 5 min, the supernatant was discarded, and 1 μL of each antibody was added to the cells (except the unlabeled tubes). A 100-µL volume of 1X PBS containing 0.05% saponin was added, and the tubes were stored at 4 °C for 30 min while protected from light. After centrifugation at 800*g* for 5 min, the supernatant was discarded by inversion, and the cells were resuspended in 300 μL of 1X PBS solution. The PBMCs were stained with mouse anti-human IL-10 conjugated to Horizon V450 (BD Pharmingen, NJ, USA).

The acquisition of 50,000 events per sample was performed in the lymphocyte quadrant (as determined by the ratio of size to granularity) using an LSRFortessa cytometer (BD Biosciences, USA). Compensation was performed using the adsorbed microspheres with anti-mouse antibodies (CompBeads, BD Biosciences, USA), and the same antibodies were used for extra and intracellular staining. The cell gating protocol was based on the specific isotype control values as well as the fluorochrome minus 1 (FMO) setting when needed. Data analysis was performed using FlowJo software (Tree Star, Ashland, OR, USA).

### Statistical analysis

The statistical analyses were performed with GraphPad Prism 5.0 (GraphPad Software Inc., La Jolla, CA, USA). The data from the in vivo and in vitro studies were obtained from three to five separate experiments with nine to 15 mice per group or 14 atopic or non-atopic individuals. Differences were considered significant when P ≤ 0.05, as assessed by Student’s *t* test and the Mann–Whitney test.

## Results

**Fig. 1:**
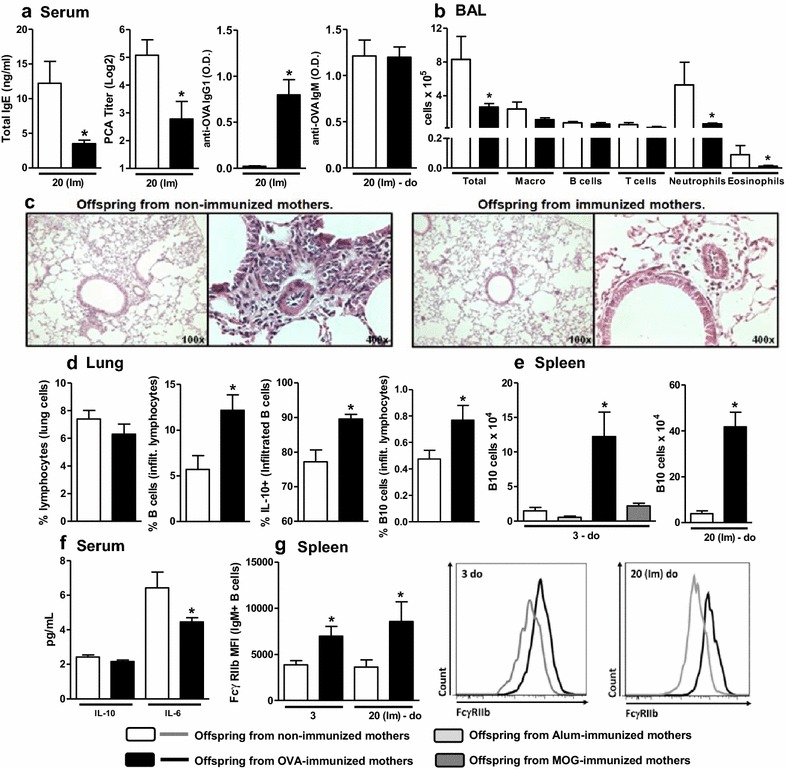
Effects of preconception immunization on offspring antibody production, lung inflammation and phenotype. Offspring from immunized or non-immunized mothers were immunized with OVA in the neonatal period and evaluated at 20 days of age [20 (Im)]. The total IgE, anti-OVA IgG1 and anti-OVA IgM levels were determined by ELISA, and anaphylactic IgE was determined by PCA (**a**). BAL preparation, histological analysis and dissociation of offspring lungs were performed in 43-day-old offspring after five intranasal challenges. The differential cell counts in BAL were evaluated by flow cytometry (**b**). Histological examinations were performed on samples stained with H&E (**c**). The dissociated lungs were evaluated by flow cytometry (**d**). The number of spleen B10 cells (**e**), the serum cytokine levels (**f**) and splenic B cell FcγRIIb expression in offspring (**g**) from Alum-, OVA- or MOG-immunized (B10 cells only) or non-immunized mothers were also evaluated by flow cytometry at 3 days of age and after OVA immunization in the neonatal period at 20 days of age [20 (Im)]. A representative histogram of FcγRIIb expression in each group is shown. The data are presented as the mean ± SEM. *P≤0.05 compared with the respective offspring from non-immunized mothers

At 20 days of age, the offspring from immunized mothers presented suppression of the total IgE and allergen-specific anaphylactic IgE titers compared with the offspring from non-immunized mothers. Moreover, increased specific IgG1 levels were detected in the offspring from immunized mothers compared with the offspring from non-immunized mothers. An evaluation of specific IgM production revealed similar levels in the offspring from immunized or non-immunized mothers after neonatal immunization (Fig. 1a). Lung inflammation in 60-day-old immunized mice was evaluated through bronchoalveolar lavage fluid (BAL) and histological analyses, and maternal immunization induced significant protection against allergen-induced airway inflammation, as evidenced by reductions in the numbers of neutrophils and eosinophils compared with the control group (Fig. 1b) and by the histological observation of a reduced cell-infiltrating profile (Fig. 1c).

We also found that the lung tissue infiltrating lymphocytes in the offspring from immunized mothers contained a higher amount of B cells that frequently produced IL-10 and expressed the B10 phenotype (CD19^+^IL-10^+^CD1d^high^; Fig. 1d) than those identified in the offspring from non-immunized mothers. A subsequent evaluation of the regulatory lymphocyte numbers on offspring spleens demonstrated that maternal OVA immunization substantially induced B10 cells in 3-day-old offspring, and this induction was maintained at 20 days of age after neonatal immunization (Fig. 1e). We also evaluated whether maternal immunization with the adjuvant Alum alone or in combination with a purified non-allergenic protein (MOG) could induce B10 cells in offspring and observed no effect (Fig. 1e). Analyses of the offspring sera revealed that maternal immunization reduced systemic IL-6 production in immunized offspring (at 20 days of age) without influencing systemic IL-10 production compared with the levels observed in offspring from non-immunized mothers (Fig. 1f). We also evaluated the impact of maternal immunization on FcγRIIb expression in offspring splenic B cells. Maternal immunization increased offspring FcγRIIb expression in IgM^+^ B cells at three and 20 days of age after neonatal immunization (Fig. 1g).

**Fig. 2:**
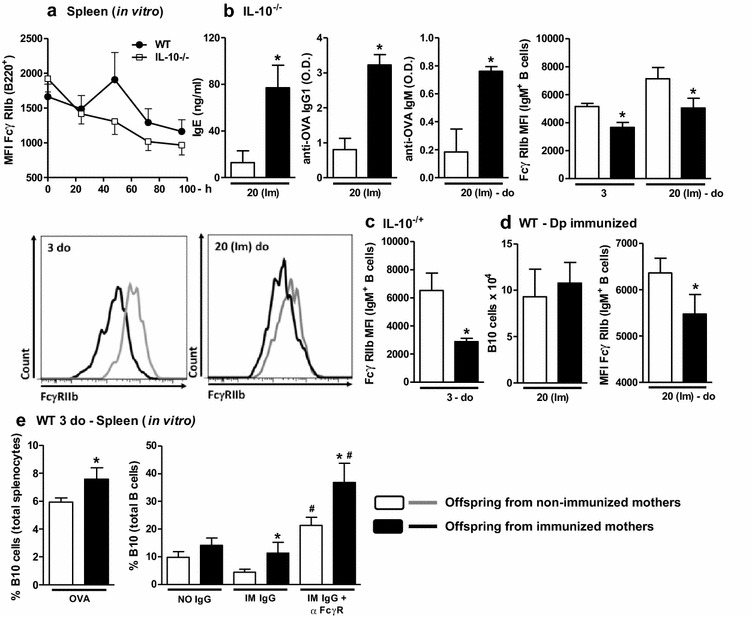
Role of IL-10 in FcγRIIb expression on B cells and in vitro effect of purified IgG. FcγRIIb expression in response to anti-IgM stimulus was assessed in WT and IL-10^−/−^ adult mice (**a**). Offspring from immunized or non-immunized IL-10^−/−^ mothers were evaluated at 3 days of age or immunized with OVA in the neonatal period and evaluated at 20 days of age [20 (Im)]. The total IgE, anti-OVA IgG1 and anti-OVA IgM levels were determined by ELISA, and splenic B cell FcγRIIb expression was evaluated by flow cytometry. A representative histogram of FcγRIIb expression in each group is shown (**b**). Identical maternal and neonatal OVA immunization protocols were performed using IL-10^−/−^ females and WT males, and splenic B cell FcγRIIb expression in IL-10^−/+^ offspring was evaluated by flow cytometry analysis (**c**). Similar maternal and neonatal immunization protocols were performed using Dp, and the splenic B10 cell numbers and B cell FcγRIIb expression in offspring were evaluated by flow cytometry (**d**). Offspring splenocytes were cultured for 7 days with 20 µg/mL OVA or 100 µg/mL purified IgG from non-immunized (NO IgG) or immunized mothers (IM IgG) with or without FcγRII/III-blocking Ab. The percentage of B10 cells was evaluated by flow cytometry (**e**). *P ≤ 0.05 compared with the offspring of the respective non-immunized mothers. ^#^ *P* ≤ 0.05 compared with the respective result from the sample without FcγR-blocking Ab

Because maternal immunization induces an increase in offspring B10 cell frequency associated with RIIb upregulation, we sought to confirm the role of IL-10 in FcγRIIb expression in normal B cells. Upon anti-IgM activation, IL-10^−/−^ B cells showed the inability to overexpress FcγRIIB in response to anti-IgM activation compared with WT B cells (Fig. 2a). Based on this observation, we sought to verify the involvement of IL-10 by subjecting IL-10^−/−^ mice to a maternal/neonatal immunization protocol and evaluating the offspring with similar parameters to WT mice. The maternal immunization of IL-10^−/−^ mice resulted in augmented levels of total IgE and specific IgG1 and IgM and decreased FcγRIIB expression in offspring B cells compared with that observed in WT mice (Fig. 2b). The effect of immunization on FcγRIIB expression in offspring B cells was maintained even in partially-IL-10-deficient hybrid mice (IL-10^−/+^; Fig. 2c). Our laboratory previously reported that the maternal *Dermatophagoides pteronyssinus* (Dp) immunization protocol induces an opposite effect on FcγRIIB expression in offspring B cells compared with OVA [13], and we thus evaluated the induction of B10 cells in this model. Even at a higher dose of allergen than that used in our previous experiments, maternal Dp immunization did not induce B10 cells in offspring, and the opposite effect on FcγRIIB expression was maintained (Fig. 2d).

We then evaluated the effects of in vitro OVA stimulation on pup splenocytes in terms of the B10 percentage. As observed ex vivo, the augmented B10 cell percentage was maintained in the splenocytes of pups from immunized mothers after culture in the presence of OVA or IgG purified from immunized mothers compared with those from non-immunized mothers. We also investigated whether the engagement of the FcγRIIB receptor is involved in the increased frequency of B10 cells observed in response to IgG using a FcγR-blocking antibody in vitro. We found that the culture of splenocytes of offspring from non-immunized mothers in the presence of immunized IgG resulted in a higher B10 cell frequency after FcγRIIB blocking compared with the frequency observed after culture without FcγRIIB blocking. Splenocytes of offspring from immunized mothers yielded an even higher B10 cell frequency after FcγRIIB blocking (Fig. 2e).

**Fig. 3:**
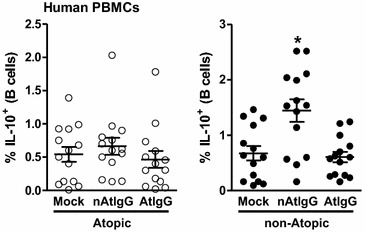
In vitro effect of human purified IgG from atopic and non-atopic individuals. PBMCs from atopic or non-atopic individuals were evaluated after 7 days of culture in the absence (mock) or presence of 100 µg/mL purified IgG from non-atopic (nAtIgG) or atopic (AtIgG) individuals, and the frequency of intracellular IL-10 in B cells was evaluated by flow cytometry. *P ≤ 0.05 compared with the mock condition

To evaluate the hypothesis that B10 cells can respond to purified IgG in humans, PBMCs from atopic and non-atopic individuals were evaluated in vitro. Using the same period and IgG concentration that were used in the murine model, we found that B cells from non-atopic individuals cultured with autologous purified non-atopic IgG presented upregulated intracellular IL-10 production compared with that detected with mock culture. This effect was not detected in response to heterologous atopic IgG. B cells from atopic patients did not produce IL-10 in response to autologous atopic IgG or heterologous non-atopic IgG compared with the mock culture (Fig. 3).

## Discussion

The maternal immunization protocol with OVA suppressed the offspring allergic response, sensitizing the offspring at a similar humoral level as previously observed by our laboratory in a similar mouse model [5, 13]. The protection induced by maternal immunization in murine models was previously evidenced by other researchers [15]. In contrast, the induction of offspring B10 cells that infiltrate more frequently into lung tissue of inhibited offspring is unedited evidence. This induction of B10 cells appears to be specific to OVA because maternal immunization with Alum alone or other non-allergenic protein (MOG) could not induce this cell population in offspring.

Both human and murine B10 cells have been shown to produce large amounts of IL-10 and to exert an inhibitory effect on allergy development. Furthermore, B10 cell activity is related to the regulation of IgE production, as demonstrated in an atopic dermatitis-like mouse model [16].

We previously used a similar preconception allergen immunization protocol with BALB/c mice to reveal that maternal immunization is associated with an upregulation of FcγRIIb in offspring B cells [7]. However, at that time, we were unable to elucidate the mechanism through which this phenotype was induced.

Using IL-10^−/−^ mice, we found that IL-10 appears to be necessary for the upregulation of FcγRIIb expression in murine B cells, as observed in WT offspring from OVA-immunized mothers. To assess the contribution of IL-10 in vivo, IL-10^−/−^ mice were subjected to a maternal and neonatal immunization protocol with OVA, which resulted in high IgE production and an opposite effect on FcγRIIb expression in offspring B cell compared with the WT animals. Furthermore, the downregulation of FcγRIIb expression was not reversed in partially-IL-10-producing offspring derived from IL-10^−/−^ mothers mated with WT males (IL-10^−/+^).

These data suggest the involvement of IL-10 in the regulation of FcγRIIb receptors on offspring B cells and that induced B10 cells can play a pivotal role in offspring as the main source of IL-10 to induce the regulatory effect of maternal immunization on offspring B cells.

The role of B10 cell induction on allergy inhibition in offspring is further reinforced by the results obtained from the neonatal Dp immunization of offspring derived from Dp-immunized mothers. The opposite effect of Dp on FcγRIIb expression in offspring B cells relative to that obtained with OVA preconception immunization was recently described by our research group [17]. In this study, we utilized a similar murine protocol with a lower Dp immunization dose and evaluated the frequency of B10 cells in offspring.

This evaluation revealed that maternal immunization with allergen extracts does not induce a specific B10 concomitantly to the reduced levels of FcγRIIb. This result suggests that the induction of B10 cells in response to allergen is important in FcγRIIb upregulation. The absence of B10 cell induction might also explain why anti-Dp IgE inhibition is not sustained after the weaning period, as we previously observed with the Dp murine model [5]. Dp is unable to induce B10 cells potentially due to the higher epitope diversity of Dp compared with that of the purified allergen OVA, but further experiments using purified allergenic proteins must be undertaken to support this hypothesis.

Since 2003, our laboratory has suggested that maternal IgG plays a role in offspring allergy inhibition that might include other mechanisms in addition to direct the avoidance of offspring sensitization due to allergen neutralization [4, 5, 7, 18, 19]. More than 15 years ago, Stadler and coworkers discussed the role of anti-idiotypic antibodies as regulators of IgE production [20]. Our in vitro results reveal that B cells of offspring from immunized mothers presented a higher B10 cell percentage in response to OVA or maternal IgG. This finding suggests an antigen-specific response by B10 cells, which engage the B cell receptor (BCR) through direct recognition of the allergen, or by allergen-specific IgG, which might idiotypically interact with BCR.

Consistent with these lines of evidence, the production of IL-10 by B10 cells was recently shown to be dependent on the BCR signaling pathway, and its function can inhibit murine allergies and autoimmunity [21]. The induction and/or maintenance of B10 cells in response to specific IgG is unprecedented in the literature and appears to be counter-regulated by FcγRIIb expression because the blockage of FcγRIIb resulted in higher levels of B10 cells even in offspring from non-immunized mothers.

In the present study, we did not evaluate whether offspring IgG also plays a role in self B10 cells, but such a role is likely because offspring IgG directly reflects the maternal immune repertoire [22] via a mechanism referred to as maternal imprinting.

Together and in general, these results suggest an allergen-specific regulatory mechanism controlled mainly by specific IgGs that are responsible for the maintenance and/or induction of allergen-specific B10 cells. The recently proposed “MatIgG primary modulation theory” describes this mechanism [23].

The production of IL-10 by B10 cells in response to allergens plays a pivotal role in allergy inhibition. Several years ago, Verhasselt and colleagues suggested that the maternal allergen-IgG immune complex transferred to offspring by breast feeding can induce tolerance in the offspring without interaction with FcγRIIb receptors [15]. Based on our results, it is possible that the induction of Breg cells could also be involved in the cited work, but the previous study only evaluated Treg cell induction.

In our model, we did not evaluate the impact of breast feeding only. Previous results obtained by our research group in a similar murine model revealed that an offspring derived from a non-immune mother and nursed by an OVA-immunized mother presents an allergen-specific anaphylactic IgE response that is suppressed at a level similar to those observed in offspring that were delivered and nursed by a OVA-immunized mother [6]. In the present work, we found that B10 cell induction can be accessed on 3-day-old offspring from immune mothers, which suggests that the proposed induction of B10 cells is mediated mainly by the transplacental transference of maternal IgG, but we cannot discard the possibility that milk IgG could also participate in the maintenance and/or induction of this mechanism.

Because this hypothesis has not been discussed in a human context, we evaluated atopic and non-atopic individuals in search of corroborating evidence and found that only peripheral B cells from non-atopic individuals can produce IL-10 in response to autologous IgG. This finding is unprecedented in the literature and suggests that B cells from non-atopic individuals have the potential to produce IL-10 in response to the IgG repertoire, as observed in our murine model. Based on our experimental evidence, we believe that this response can aid the maintenance of a non-atopic immune status. The protective effect of maternal passively transferred IgG induced by allergen immunotherapy in humans has been described in the literature [24, 25], but a possible mechanism for B10 cell induction in humans has not been described.

## Conclusions

In conclusion, our results reveal that maternal immunization with allergens can induce B10 cells that respond to allergen or allergen-specific IgG. This response is unprecedented and occurs in mice and humans, revealing a possible mechanism for allergy inhibition that will yield new targets for the future development of allergy inhibition protocols.

## Additional file

**Additional file 1: Figure S1.** Illustrative dot plots of gating strategy to identify B cells that produces IL-10 and that express B10 phenotype on offspring spleen. Each sample was acquired in the singlet cells gate (determined by FSC-A/FSC-H parameters), panels represent gate strategy then in the lymphocytes gate (determined by their relative size/granularity), and then gated as CD19+ cells (B cells), IL-10 (IL-10+B cells) and CD1d^high^ cells (B10 cells).

## Abbreviations

B10: B regulatory cells that produce IL-10
OVA: ovalbumin
Breg: B regulatory cells
WT: wild type
PBMCs: peripheral blood mononuclear cells
SPT: skin prick test
BAL: bronchoalveolar lavage fluid
Dp: *Dermatophagoides pteronyssinus* mite
BCR: B cell receptor
