# Supplementary material for: Preconception allergen sensitization can induce B10 cells in offspring: a potential main role for maternal IgG
Source: Allergy Asthma Clin Immunol. 2017 Apr 17;13:22. doi: 10.1186/s13223-017-0195-8 (PMC5392917; doi:10.1186/s13223-017-0195-8)
Supplement: Supplementary file 1 — Additional file 1: Figure S1. Illustrative dot plots of gating strategy to identify B cells that produces IL-10 and that express B10 phenotype on offspring spleen. Each sample was acquired in the singlet cells gate (determined by FSC-A/FSC-H parameters), panels represent gate strategy then in the lymphocytes gate (determined by their relative size/granularity), and then gated as CD19+ cells (B cells), IL-10 (IL-10+B cells) and CD1dhigh cells (B10 cells). [file 13223_2017_195_MOESM1_ESM.pdf]

Supplementary material

Figure S1

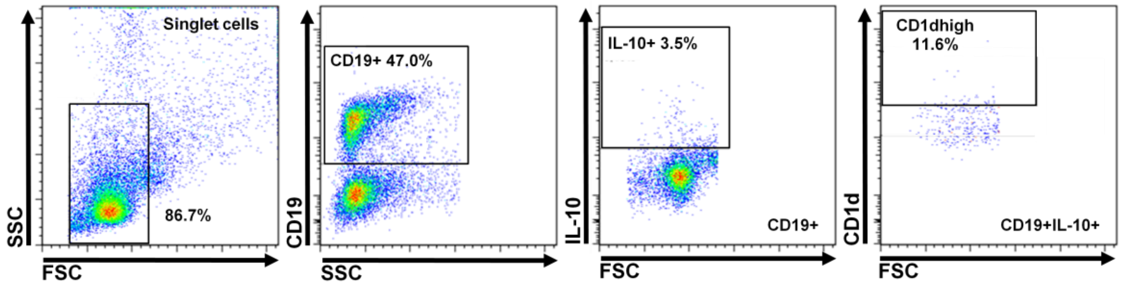

Figure S1: Illustrative dot plots of gating strategy to identify B cells that produces IL-10 and that express B10 phenotype on offspring spleen. Each sample was acquired in the singlet cells gate (determined by FSC-A/FSC-H parameters), panels represent gate strategy then in the lymphocytes gate (determined by their relative size/granularity), and then gated as CD19+ cells (B cells), IL-10 (IL-10+ B cells) and CD1d^{high} cells (B10 cells).
